# Two new ootaxa from the late Jurassic: The oldest record of crocodylomorph eggs, from the Lourinhã Formation, Portugal

**DOI:** 10.1371/journal.pone.0171919

**Published:** 2017-03-08

**Authors:** João Russo, Octávio Mateus, Marco Marzola, Ausenda Balbino

**Affiliations:** 1 GeoBioTec, Faculdade de Ciências e Tecnologia, Universidade Nova de Lisboa, Caparica, Portugal; 2 Museu da Lourinhã, Lourinhã, Portugal; 3 Department of Geosciences and Natural Resource Management, University of Copenhagen, Copenhagen, Denmark; 4 Departamento de Geociências, Universidade de Évora, Évora, Portugal; University of Akron, UNITED STATES

## Abstract

The Late Jurassic Lourinhã Formation is known for its abundant remains of dinosaurs, crocodylomorphs and other vertebrates. Among this record are nine localities that have produced either dinosaur embryos, eggs or eggshell fragments. Herein, we describe and identify the first crocodiloid morphotype eggs and eggshells from the Lourinhã Formation, from five occurrences. One clutch from Cambelas, composed of 13 eggs, eggshell fragments from Casal da Rola and Peralta, one crushed egg and eggshells from Paimogo North, and four crushed eggs as well as eggshell fragments from Paimogo South. We observed and confirmed diagnostic morphological characters for crocodiloid eggshells and which are consistent with a crocodylomorph affinity, such as the ellipsoidal shape, wedge-shaped shell units, triangular extinction under cross-polarized light, and tabular ultrastructure. This material is distinctive enough to propose two new ootaxa within the oofamily Krokolithidae, *Suchoolithus portucalensis*, oogen. and oosp. nov., for the material from Cambelas, the most complete clutch known for crocodiloid eggs, and *Krokolithes dinophilus*, oosp. nov., for the remaining material. These are the oldest crocodylomorph eggs known, extending the fossil record for this group to the Late Jurassic. Furthermore, except for the clutch from Cambelas, the material was found with theropod eggs and nests, in the other four occurrences, which seem to suggest some form of biological relationship, still unclear at this point.

## Introduction

**Fig 1:**
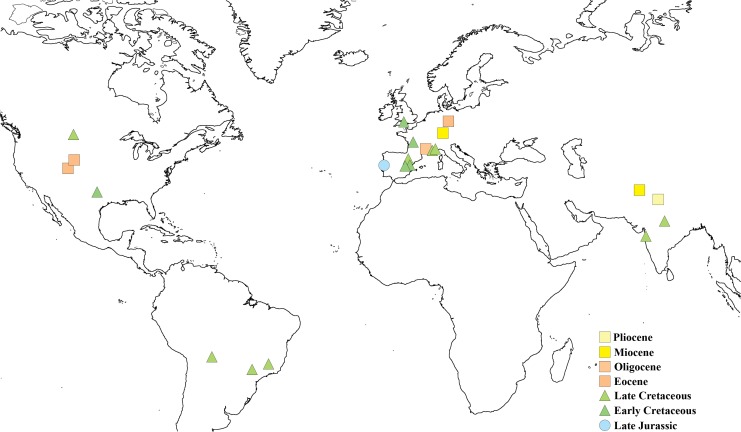
Geographic distribution of fossil eggs and eggshells ascribed to Crocodylomorpha.

Today Crocodylomorpha are represented by 24 species of Crocodylia, a group that originated within Eusuchia during the Late Cretaceous [1,2]. Although the extant diversity is low, the fossil record of crocodylomorphs is extensive, dating to the Late Triassic, with numerous different forms and a much more diverse ecological distribution [1,3–6]. Fossil eggs of Crocodylomorpha are still scarce and poorly understood, even though occurrences of eggshells attributed to the group have been identified worldwide ([7–12], see also Fig 1 and S1 Table). The rarity of eggshells attributed to the clade stands out when compared to the number of occurrences of fossilized eggs of other groups, namely dinosaurs. Eggshells of the crocodiloid basic type, which includes only one morphotype, defined by [13,14], and exclusively identified in fossil and extant Crocodylomorpha, show remarkably low morphological variation in their structure [8,15]. The set of distinctive structural characters consistently observable throughout the fossil record and in the extant representatives of the group allow for a conclusive taxonomical assignment.

The first descriptions of crocodilian eggshells date back to the 30’s and 40’s of the twentieth century [16,17]. However, it was the studies by Erben and colleagues [18,19] decades later, that first used scanning electron microscope (SEM) data and applied the concept of biomineralization to eggshell structure description. These works described and identified the biomineralogical organization and diagnostic characters of eggshells, and established relationships between the major amniote groups, including Crocodylomorpha, and specific morphological eggshell arrangements, providing a more solid framework for later paleontologists [13,14,20–27].

### Systematics of crocodiloid eggshells

The great diversity of fossil eggshells prompted Mikhailov [28] to advocate and propose a strictly parataxonomical system to classify fossil eggs, following the work initiated by Zhao [29]. The nomenclature should use the general rules of the International Commission on Zoological Nomenclature (ICZN) as applied to ichnotaxa. Such a classification had already been used though by Hirsch [26] to erect the crocodiloid (*sensu* [13]) oogenus *Krokolithes* and the oospecies, *Krokolithes wilsoni*, based on the micro- and ultrastructure observed in eggshells from the DeBeque Formation (Eocene) of Colorado and of extant crocodiles. The oofamily Krokolithidae was named by Kohring and Hirsch [23] who at the same time included a second oospecies within *Krokolithes*, *K*. *helleri*. A second oogenus, *Bauruoolithus fragilis*, within Krokolithidae was erected by Oliveira and colleagues [7], from the Late Cretaceous Adamantina Formation of Brazil, although it was recently recognized as a *nomen nudum* by Jackson and Varrichio [30]. Recently, Moreno-Azanza and colleagues [31] described and identified material from the Early Cretaceous of Spain, and reassigned previously reported eggshells from the Purbeck of England [32,33], proposing a new oogenus and oospecies, *Mycomorphoolithus kohringi*, just outside the oofamily Krokolithidae, as *incertae sedis*, as the eggshells present a combination of features that suggest a crocodylomorph affinity, but not enough to conclusively ascribe them to Krokolithidae. Two unnamed oospecies in *Krokolithes* are known from the Early Cretaceous of Spain [8,31]. Finally, Krokolithdae indet. eggshells have been reported in the Maastrichtian of Northern Spain. Recently, Jackson and Varrichio [30] revised and emended the diagnosis of the oofamily Krokolithidae.

### Fossil record of crocodiloid eggshells

Fossil crocodiloid eggshells have been found all over the world, except in Antarctica and Australia (Fig 1). In Europe, eggshells are reported from the Lower Miocene of Ulm and the Middle Eocene of Geiseltal, Germany [10,16,23,34], from the Upper and Lower Cretaceous of France and Spain [31,32,35–43], and from the top of the Lulworth Formation (Berriasian) of the Purbeck Limestone Group (Wealden) of England [32,33]. In North America, eggshells were found in the Middle Eocene DeBeque and Bridger Formations, from Colorado and Wyoming respectively [23,26], in the Upper Cretaceous Two Medicine, Hell Creek and Fruitland Formations, from Montana and New Mexico respectively [44–46], and in the Lower Cretaceous (Albian) Glen Rose Formation from Texas [47]. It is worth mentioning that Erickson [48] described a probable crocodilian egg from the Upper Cretaceous of Wyoming, but Hirsch and Kohring [49] consider that identification highly doubtful based on the inner filling of the specimen that rather suggests a calculus. Hirsch [50] mentions very badly preserved, highly uncertain crocodilian-like eggshells from the Upper Jurassic Morrison Formation that “[…] show large shell units with indications of wedge-like structures similar to those in crocodilian eggs […] the extinction pattern is also similar to that seen in crocodilian eggs. However, before a final identification is made, the specimens must be studied in more detail”, which have never been studied in detail since. In South America, crocodylomorph eggshells are reported from the Upper Cretaceous Araçatuba and Adamantina Formations of Brazil [7,51,52] and Cajones Formation of Bolivia [53]. In Asia, Patnaik and Schleich [54] report crocodiloid eggshells from the Pliocene in the Upper Siwaliks of India. In the Upper Miocene of the Chinji Formation from Pakistan, a complete crocodylomorph egg was described by Panadés I Blas and Patnaik [55]. Crocodilian eggshells were found also in the Upper Cretaceous Intertrappean Beds, India [11,12,56].

**Fig 2:**
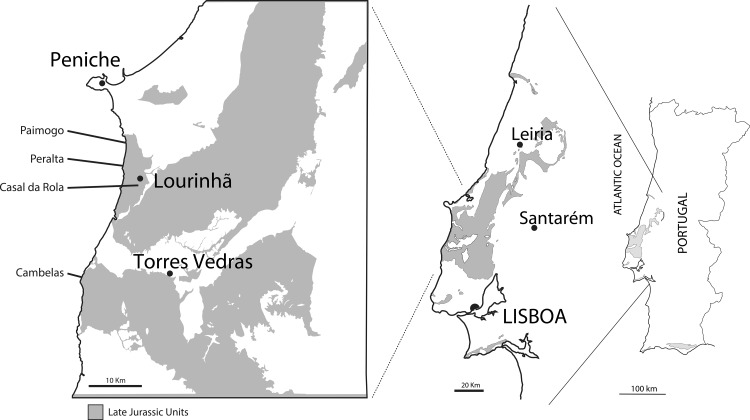
Map of the Lourinhã region, Western Portugal. Schematic geological map showing the fossil sites within the Lourinhã Formation. Late Jurassic rocks in gray. Sites and specimens: Paimogo N: ML760; Paimogo S: ML1795; Casal da Rola: ML1194; Peralta: ML159; Cambelas: FCT-UNL706. Based after [57,65].

Here we add to this record by providing a detailed re-description and interpretation of Late Jurassic crocodylomorph eggs and eggshells from five localities (Fig 2), reported by [57], four of which previously known for dinosaur eggshells and nests, Paimogo (North and South), Peralta, and Casal da Rola [57–64], making these the oldest occurrences of crocodyloid eggs known We thoroughly review and improve on previous works [57,58], adding new data that allows us to erect two new ootaxa while at the same time providing new insights into the evolution of crocodylomorph-ascribed eggshells.

## Material and methods

### Geological and paleontological setting

**Fig 3:**
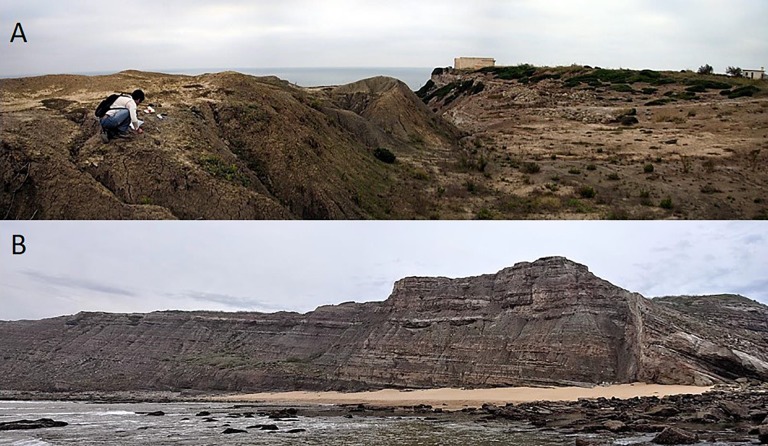
Outcrops of Lourinhã Formation. **A**, location of Paimogo, Northern Lourinhã Formation, Praia da Amoreira-Porto Novo and Praia Azul Members. **B.** location of Cambelas, Southern Lourinhã Formation, Assenta Member.

The Lourinhã Formation (Fig 2) is a massive continental depositional sequence, punctuated by some shallow marine intercalations, informally defined by Hill [66]. It is a thick syn-rift siliciclastic succession, ranging from 200 to 1100 meters in thickness, late Kimmeridgian-earliest Berriasian in age [67,68], that was deposited during the third rifting episode of an extensional event related to the opening of the North Atlantic that climaxed during the Late Oxfordian-Early Kimmeridgian [66,69,70]. The exact lithostratigraphy of the Lourinhã Formation is complex, and there is no consensus regarding its formal lithostratigraphical units. Here we use the most recent stratigraphy, defined by Mateus et al. [67]. Thus, the Lourinhã Formation is comprised of three members, from bottom to top: i) the Praia da Amoreira-Porto Novo Member (Fig 3A), ii) the Praia Azul Member, and iii) the Assenta Member (Fig 3B). The Praia da Amoreira-Porto Novo Member shows characteristics of tide-influenced upper delta, floodplain, and alluvial facies, and is interpreted as being of latest Kimmeridgian [67]. The Praia Azul Member is a mainly marl-mudstone unit with few sandstone levels, and contains three marly-carbonate shallow marine layers, indicative of brief yet relevant transgressive episodes that allow for a more precise biostratigraphical dating than in other units in the Lourinhã Formation, being latest Kimmeridgian-earliest Tithonian [67,71]. The topmost Assenta Member is dominated by mudstones, often with levels of pedogenic carbonate concretions, or caliche, evidence of paleosoils (either forming high resistance levels or the reworked nodules forming conglomerates at the base of channels), intercalated with channelized cross-bedded sandstones, including large scale point-bars, and thin flat lenses or tabular crevasse and levee bodies, and representing the late Early Tithonian to earliest Berriasian [67,68,71].

The fluvio-deltaic paleoenvironment of the Lourinhã formation created the conditions for the existence of a highly diversified ecosystem, with remarkable faunal similarities with the coeval Morrison Formation, seemingly indicating a close yet complex paleobiogeographical relationship [65]. A case in point is the dinosaur fauna [65,72–77]. Therefore, both areas would be a favorable breeding ground for a variety of organisms, such as turtles, dinosaurs, and crocodylomorphs. Despite this, the fossil egg record is comparatively scarcer in Morrison than in the Lourinhã formation. In the Late Jurassic of Portugal there are now nine known localities with reported eggs and eggshells, eight of which are located in the Lourinhã Formation, where the dinosaur fossil egg and embryo record is well documented [59–64,78–84]. There are also testudinoid eggshells reported from the Guimarota coal mine, in Leiria (70 km N of Lourinhã), from the Alcobaça Formation [85,86]. The eggs and eggshells reported in this study add to the fossil egg record from the Late Jurassic, and from the Lourinhã Formation, and more importantly, to the crocodylomorph fossil oodiversity.

### Material studied

**Fig 4:**
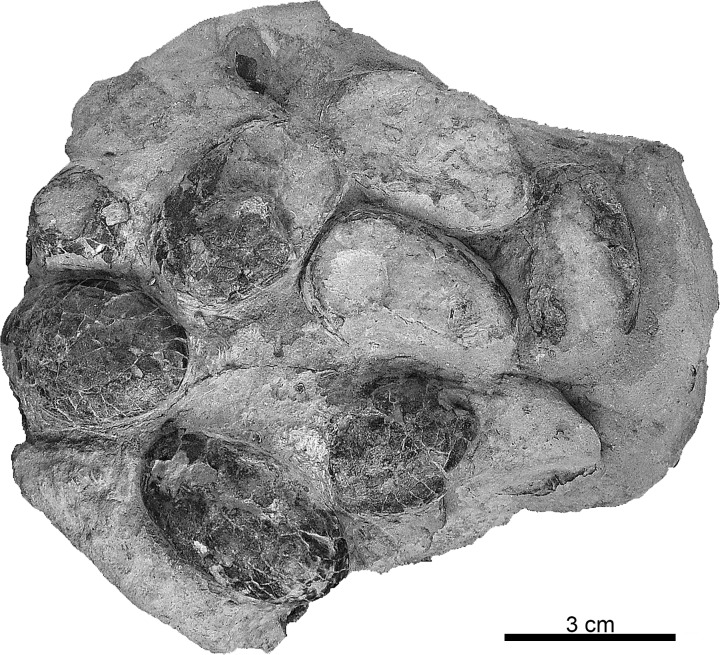
Holotype of *Suchoolithus portucalensis*, oogen. and oosp. nov. Specimen FCT-UNL706 from Cambelas, Assenta Member, Lourinhã Formation, Upper Jurassic. The shape and preservation of the specimen suggests an unhatched clutch.

**Fig 5:**
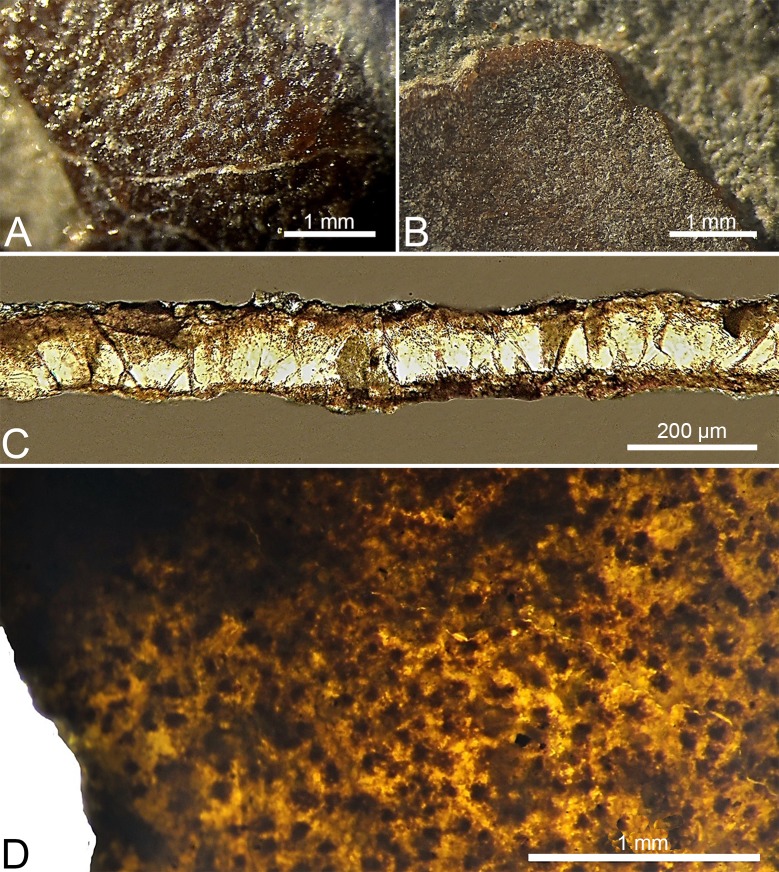
**Macro photographs (A, B) and micro photographs of *S*. *portucalensis* (C, D). A**, external surface of the eggshell; **B**, internal surface of the eggshell; **C**, radial section under petrographic microscope, with cross-polarized light; **D**, tangential section of the eggshell under stereomicroscope, with transmitted light, where the tips of the individual shell units (dark dots) are observable.

The studied material has been found and collected between 1987 and 2012 from five localities in the Lourinhã Formation (Fig 2). The specimen from Cambelas was recovered by Octávio Mateus on July 13^th^ of 2008, and is catalogued at Faculdade de Ciências e Tecnologia da Universidade Nova de Lisboa (FCT-UNL; Caparica, Portugal), catalogue number FCT-UNL706, and currently stored at Museu da Lourinhã (ML; Lourinhã, Portugal). FCT-UNL706 (Figs 4 and 5) is the only preserved clutch, on a fine gray sandstone block, with 13 eggs. Currently, and after the present study was carried, the clutch has been dismantled, but a cast is housed at ML, specimen catalogue number ML1582.

**Fig 6:**
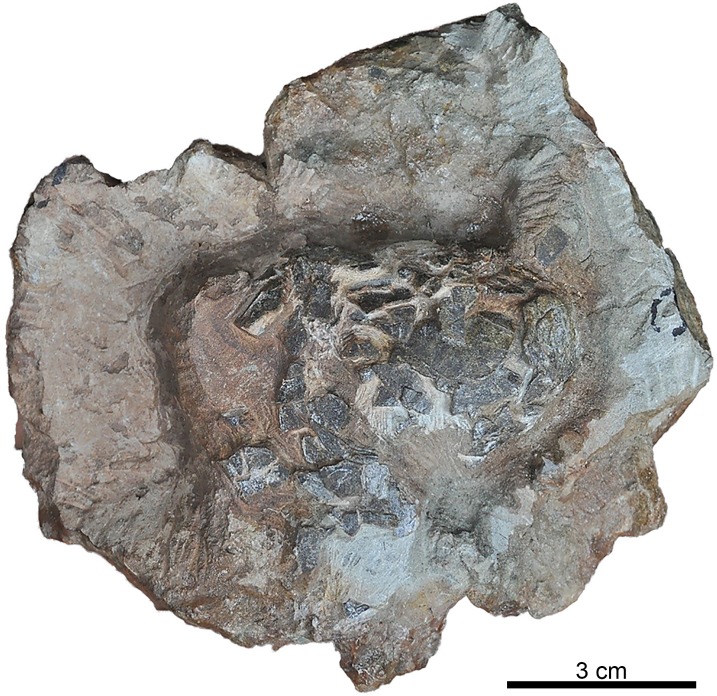
Holotype of *Krokolithes dinophilus*, oosp. nov. Specimen ML760 from Paimogo N, Praia da Amoreira-Porto Novo Member, Lourinhã Formation, Upper Jurassic.

**Fig 7:**
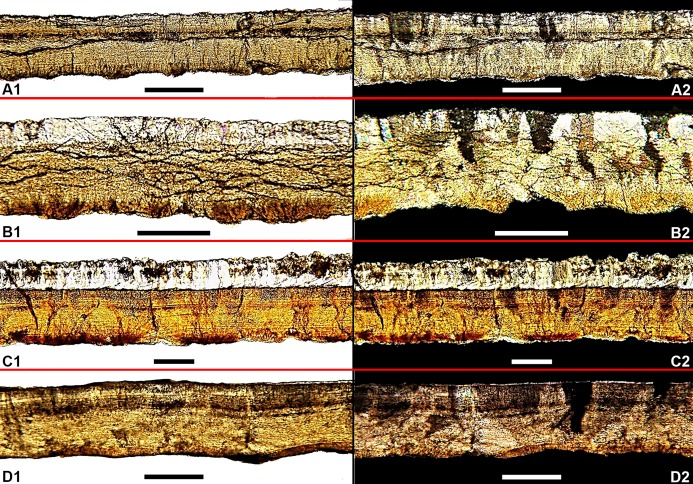
Radial sections of *K*. *dinophilus*. Right: parallel polarized light; Left: cross-polarized light. **A**, ML195; **B**, ML760; **C**, ML1795; **D**, ML1194. Scale bars: 200 μm.

**Fig 8:**
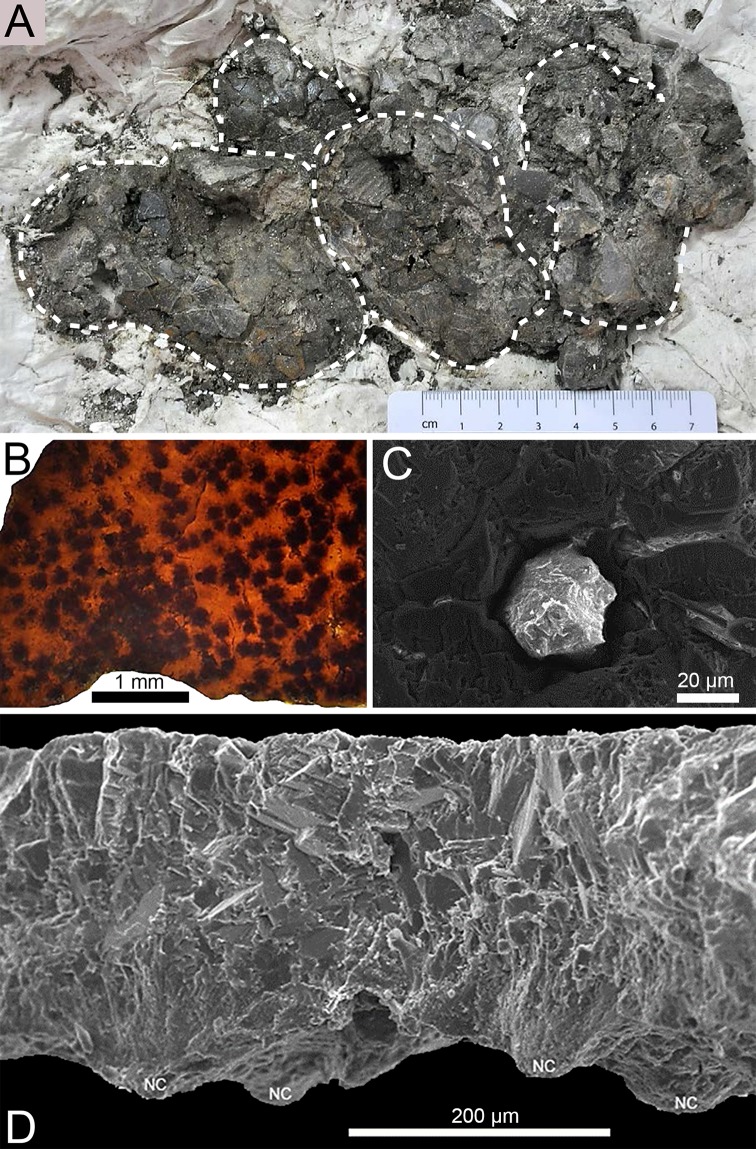
Eggs, SEM and tangential section of *K*. *dinophilus* ML1795. **A**, block with specimen ML1795, dashed white lines outlining the crushed eggs; **B**, tangential eggshell section under stereomicroscope, with transmitted light, showing the darkened mammillae tips (nucleation centers); **C**, SEM image of the external opening of a filled pore; **D**, SEM image of a transversal section of the eggshell. In **D**, the nucleation centers or basal knobs (**NC**) are evident.

**Fig 9:**
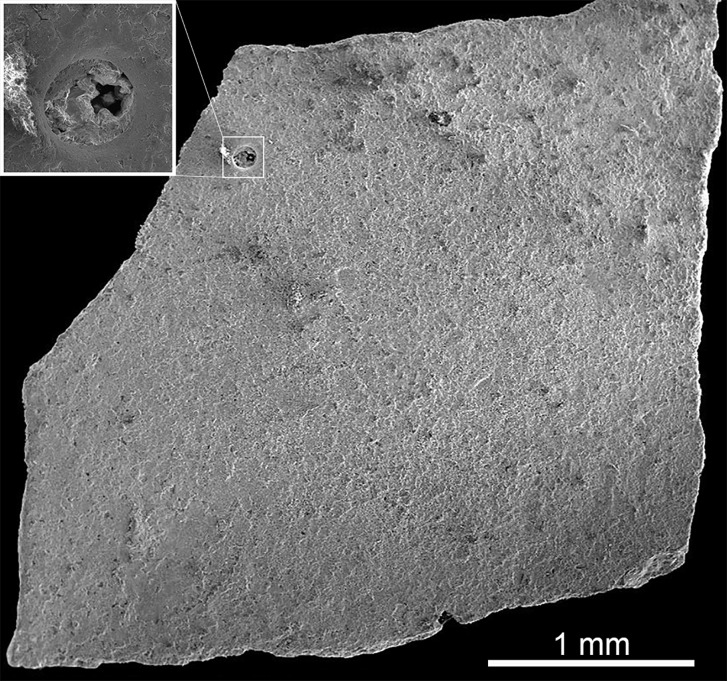
SEM photograph of *K*. *dinophilus* (ML1194). External surface of an eggshell fragment with the detailed inset of a pore opening.

From the other sites, the specimens are catalogued and stored at ML. ML760 (Paimogo North, Figs 6 and 7B) and ML1795 (Paimogo South, Figs 7C and 8) were found by Isabel Mateus and collected between 1993 and 1997 (see [58,59] for further details) by a team led by Isabel Mateus and Horácio Mateus. ML1194 (Casal da Rola, Figs 7D and 9) was found by Vasco Ribeiro and collected in 2012 by Vasco Ribeiro, Femke Holwerda, João Russo, and Emanuel Tschopp. ML195 (Peralta, Fig 7A) was found by Horácio Mateus in 1987 and collected during various excavations through the years, the last one dating from 2011. Five partial crushed eggs were recovered, all of them from the Paimogo localities. ML760 (Fig 6) is a single egg in a reddish-gray mudstone while ML1795 (Fig 8) is a dark brown mudstone block with four crushed eggs. It should be noted that in this case, due to its fragility, the specimen was left in the plaster jacket to protect its integrity. The rest of the material is the most abundant and consists of more than 200 mostly weathered, very small fragments (less than 25 mm^2^). The fragments were found loose and were either collected at the surface or by sieving sediment from the sites. From each locality, well preserved samples were selected and cleaned using an ultrasound bath, were embebed using EpoThin resin and hardener, mixed in a proportion of 5:2. and were thin sectioned in radial sections and observed with a petrographical microscope (Labomed CXL POL). Macro photographs under a Leica MZ6 stereomicroscope were also taken of the outer and inner surface of the eggshells, to observe the pores and the distribution of the shell units. A stereomicroscope with transmitted light has been used with tangential eggshell sections in order to identify, when possible, the distribution of shell units, mammillae and nucleation centers, and detect the presence and shape of pores, as previously done with extant crocodilian eggshells [9]. The observations under the petrographic and stereomicroscopes were done at ML and FCT-UNL. SEM imaging was done at FCT-UNL using a JEOL JSM T330A scanning electron microscope and at Universidade de Évora (UE; Évora, Portugal) using a Hitachi SN-3700 scanning electron microscope. When referring to the eggshell total thickness and ratios between layers, we do not include the diagenetic layer. No permits were required for the described study, which complied with all relevant regulations.

### Nomenclatural acts

The electronic edition of this article conforms to the requirements of the amended International Code of Zoological Nomenclature, and hence the new names contained herein are available under that Code from the electronic edition of this article. This published work and the nomenclatural acts it contains have been registered in ZooBank, the online registration system for the ICZN. The ZooBank LSIDs (Life Science Identifiers) can be resolved and the associated information viewed through any standard web browser by appending the LSID to the prefix “http://zoobank.org/”. The LSID for this publication is: urn:lsid:zoobank.org:pub:C8E78057-9BEA-4B55-85A5-44D14778294C. The electronic edition of this work was published in a journal with an ISSN, and has been archived and is available from the following digital repositories: PubMed Central, LOCKSS [author to insert any additional repositories].

## Results

### Parasystematic paleontology

KROKOLITHIDAE Kohring and Hirsch, 1996 [24]

*Suchoolithus portucalensis* oogen. et oosp. nov.

ZooBank Life Science Identifier (LSID) for the oogenus: urn:lsid:zoobank.org:act:C5B99229-C8C5-4322-B7B7-9E827DBF8F3B

#### Type oospecies

*Suchoolithus portucalensis* oosp. nov

#### Etymology

*Suchoolithus* derives from *suchus*, the latinized Greek word for “crocodile”, and Greek *oolithus* means “egg stone”.

#### Diagnosis

The same as for the oospecies.

*Suchoolithus portucalensis* sp. Nov

ZooBank LSID for the oospecies: urn:lsid:zoobank.org:act:3C59EF39-CBA2-4C91-A91A-DDFDA65F6A95

#### Etymology

The specific epithet”*portucalensis*” refers to Portugal, the country of origin.

#### Holotype

FCT-UNL706, a clutch with 13 eggs (replica ML1582 stored at Museu da Lourinhã).

#### Diagnosis

As combined characters: ellipsoid eggs, size approximately 42 x 26 mm; ornamented outer surface with very small bumps; average shell thickness of 160 μm; trapezoidal shell units tightly packed together and wider than taller, with almost no interstices at the bases of the shell units.

#### Locality and horizon

39° 04’ 58,84” N; 9° 25’ 01,58” W, Cambelas, Torres Vedras, Portugal. Assenta Member, Lourinhã Formation, upper Tithonian, Upper Jurassic.

#### Description

Macroscopically, the holotype FCT-UNL706 is a clutch with 13 eggs (Fig 4), seven of which are well preserved and mostly intact. The remaining are incomplete, composed of aggregates of eggshell fragments *in situ*. Some of the eggs are truncated, either by erosion or excavation of the clutch or by hatching, although the undisturbed aspect of the clutch allows us to consider the first scenario as the most likely. It was found in a fallen block of fine sandstone, and no sedimentological features of the block allow polarity orientation, other than the truncation of eggs, where the truncation of upper halves of the egg is more probable. Considering this orientation, most of the eggs are shown in the bottom part of the specimen, with three of the eggs only visible on the upper of the clutch. Except for two eggs, which are oriented vertically, all the others are oriented horizontally. The eggs are dark brown, standing out from the very fine, light gray sandstone matrix, and show a fractured and cracked surface. Nonetheless, the clutch is well preserved and, even though there is truncation in some of the eggs which is a form of fossil diagenetic alteration, the eggs do not show any signs of any other severe post-burial disarrangement. Furthermore, there is no evidence of recrystallization or replacement of the original composition of the eggs.

The eggs are ellipsoid in shape, with blunt ends, measuring approximately 42 mm long and 26 mm wide, with an elongation index (EI) of 1.62. The external surface is lightly sculptured by slightly uneven, tiny bumps (Fig 5A). No evidence of extrinsic degradation can be appreciated. The internal surface shows a tight packing of the basal knobs (Fig 5B). No pore openings have been observed.

The shell thickness is 160 μm (n = 80, sd = 17 μm). Microscopically, in radial section, the wedges of the shell units are clearly visible, interlocked and closely packed together with little space between them, with a wider top and gradually narrowing until the darker basal knobs (Fig 5C). A very thin, discontinuous diagenetic layer covers the outer surface. No growth lines are visible. The basal plate groups are present (Fig 5C), although not observable through the whole radial section. There is a very thin, darker line at two thirds of the eggshell thickness. The shell units are sometimes domed in the upper part, resulting in the bumpy outer surface ornamentation, and a blocky extinction pattern is present when observed under cross-polarized light (Fig 5C). In Fig 5D, with transmitted light, on a tangential section, darker areas, corresponding to the basal plate groups, are clearly visible and show a distribution identical to what is observed in extant crocodyloid eggs [9].

*Krokolithes* Hirsch 1985 [26]

#### Type oospecies

*Krokolithes wilsoni* Hirsch 1985 [26].

#### Holotype

UCM 47523A/HEC 93, one of four eggs from a clutch [26].

#### Type locality

UCM Locality 81079, near Parachute, Garfield County, Colorado, USA. DeBeque Formation, Eocene [26].

#### Referred oospecies

*Krokolithes wilsoni* Hirsch 1985 [26]; *Krokolithes helleri* Kohring and Hirsch 1996 [23]; *Krokolithes dinophilus* sp. nov.

#### Diagnosis

Diagnosis *sensu* [46] and amended to include the thinner eggshells described in this study (as combined characters): eggshell with outer surface smooth to undulating; straight pore canals ending between shell units in deep interstices; ellipsoidal eggs with two blunt ends; egg size 68–50 mm and 44–30 mm, shell thickness 170–760 μm.

*Krokolithes dinophilus* oosp. nov.

ZooBank LSID for the oospecies: urn:lsid:zoobank.org:act:6E689451-1BA1-495C-B063-E7BD2D6DAED0

#### Etymology

The epithet “dinophilus” refers to the occurrence of these eggshells with dinosaur nests and eggshells.

#### Holotype

ML760, one crushed egg.

#### Type locality and horizon

Paimogo, Lourinhã, Portugal. Top of the Praia da Amoreira-Porto Novo Member, Lourinhã Formation, uppermost Kimmeridgian, Upper Jurassic.

#### Referred material

ML195, less than 20 eggshell fragments; ML1194, between 10 and 30 eggshell fragments; ML1795, four crushed eggs and between 150 and 200 eggshell fragments.

#### Locality and horizon

ML760, Paimogo North, Lourinhã, Portugal. Top of the Praia da Amoreira-Porto Novo Member, Lourinhã Formation, uppermost Kimmeridgian, Upper Jurassic; ML1795 Paimogo South, Lourinhã, Portugal. Base of the Praia Azul Member, Lourinhã Formation, uppermost Kimmeridgian, Upper Jurassic; ML1194, Casal da Rola. Praia Azul Member, Lourinhã Formation, uppermost Kimmeridgian-lowermost Tithonian, Upper Jurassic; ML195, Peralta, Lourinhã, Portugal. Praia Azul Member, Lourinhã Formation, uppermost Kimmeridgian-lowermost Tithonian, Upper Jurassic.

#### Diagnosis

Large *Krokolithes* eggs, approximately 70 x 40 mm, with thin eggshells (170–250 μm), strongly pronounced growth lines (horizontal lamination), narrow trapezoidal shell unit, with small interstitial space between their bases.

#### Description

**ML760**. Macroscopically, ML760 is a crushed egg (Fig 6) found in association with a theropod nest [58]. The egg is encased in a small block of reddish mudstone with some caliche nodules. Even though crushed, the egg retains a characteristic ellipsoid shape with blunt ends. It measures 70 mm in length and 40 mm in width, with an EI of 1.75. The external surface is smooth and the dark gray shell is very fractured. No pores were identified in a macroscopic observation.

Eggshell thickness is 248 μm (n = 80, sd = 14 μm). Microscopically, in radial section, (Fig 7B), the basal knobs and nucleation centers are evident, but the trapezoidal shell units are faint and in most cases, hard to define due to a strong sub-horizontal fracturing that prevents a clear observation of the tabular growth structure which is barely visible (Fig 7B). The basal plate groups make up approximately 20% of the eggshell thickness. A diagenetic layer, with a thickness of 71 μm, of diagenetic secondary deposits of calcite and recrystallization, covers the external surface. The eggshell has an extremely low porosity (less than one pore per cm^2^). In cross-polarized light (Fig 7B2), the irregular triangular extinction pattern is clearly visible.

**ML195.** ML195 are small, dark gray, eggshell fragments, less than 25 mm^2^ each, also found in association with a theropod nest. The outer and inner surfaces are smooth, with no discernible internal bumps of the basal knobs or pore openings on direct observation.

The eggshell thickness is 250 μm (n = 80, sd = 8 μm), with a 14 μm diagenetic layer overlaying the outer surface. Under the microscope, the radial section (Fig 7A) shows the darker basal knobs align along the inner surface of the eggshell, about 30 μm thick (approximately 14% of the total shell thickness). The trapezoidal shell units are very faint and barely distinguishable, but still present. The horizontal lamination or tabular structure is unevenly distributed, with a lighter colored portion and fainter growth lines just above the basal knobs (Fig 7A) making up about 160 μm of the total shell thickness (approximately 62%). Just above it, there is a thin darker band of more compacted horizontal growth lines (Fig 7A), approximately 60 μm thick (about 24% of the shell thickness). In cross-polarized light, the blocky extinction is present (Fig 7A2), although less conspicuous than in ML760, ML1795 and ML1194.

**ML1194.** Macroscopically, ML1194 are small fragments, also found in association with theropod eggs [64], very similar to ML195, both in dimensions and morphology. The inner and outer surfaces are smooth. In macroscopic observation, pores were not observed.

Eggshell thickness is 220 μm (n = 80, sd = 7 μm). Microscopically, in radial section, the shell units (Fig 7D) are very faint and hard to distinguish. The basal knobs and nucleation centers make up approximately 16% of total shell thickness, with 35 μm, and are characterized by a darker coloration (Fig 7D). About 140 μm thick (approximately 63% of shell thickness), there is a portion of the eggshell characterized by a horizontal tabular lamination that shows an increase inits density from the bottom to the top (Fig 7D). A darker zone is visible (Fig 7D) just above the previous, with the tabular growth more evident, more tightly packed together, about 50 mm thick (approximately 21% of total shell thickness). The diagenetic layer is very thin and sparse, not observable throughout the whole section, and at most 20 μm thick. With cross-polarized light (Fig 7D2), the extinction triangles are visible. Pores are very few (less than a pore per cm^2^), and have a subcircular opening, with a diameter of 110 μm, and straight long canals (Fig 9).

**ML1795.** ML 1795 includes four crushed, very fragmented eggs (Fig 8A), and eggshell fragments, found slightly south and above of ML760, and associated with a theropod nest [58]. Because the specimen is so fragile, it is still partially encased in its plaster jacket. Some of the dark brown mudstone matrix is present (Fig 8A). As with ML760, ML195, and ML1194, pores are undistinguishable macroscopically.

Eggshell thickness is 250 μm (n = 80, sd = 15 μm). In microscopic observation, the shell unit wedges are clearly observable, with basal knobs and nucleation centers visible (Fig 7C), and measuring about 60 μm (approximately 25% of shell thickness). The horizontal tabular lamination is present in the middle portion of the eggshell (Fig 7C), about 100 μm thick (approximately 40% of shell thickness). A darker area, just above the latter layer, showing a more compact lamination (Fig 7C), can be differentiated (approximately 90 μm thick, about 35% of shell thickness). A diagenetic layer (Fig 7C), about 140 μm, covers the external surface of the eggshell. Pores are very scarce (less than one per mm^2^) and hard to observe. Still, in Fig 8C, an obstructed pore opening can be seen. The pore diameter is 42 μm. The internal openings are not visible in the samples. With cross-polarized light, the irregular triangular extinction is observable (Fig 7C2). Under the stereomicroscope and using transmitted light, the darker areas corresponding to the basal knobs and tips of the shell units are evident in the tangential section (Fig 8B). Under the SEM, the nucleation centers are noticeable (Fig 8D).

### Comparison with crocodylomorph eggshells

The material presented in this study is distinct from other occurrences of fossil crocodylomorph eggshells, namely in size and eggshell thickness (see S1 Table), in the distribution of horizontal accretion lines and wider, tightly packed shell units (Fig 8). Morphologically, *S*. *portucalensis* is clearly crocodiloid, namely the ellipsoid shape, the external ornamentation, the trapezoidal shell units, the extinction pattern in cross-polarized light. However, the eggs are much smaller than in *Krokolithes*. Also, the shell units in *S*. *portucalensis* are much more packed together and the interstices are almost absent. The shell thickness (≈ 160 μm) of *S*. *portucalensis* is one of the lowest in the fossil record, and only eggshells described by Oliveira et al. [7] are slightly thinner (≈ 150 μm).

The eggs of *K*. *dinophilu*s are slightly larger (70 x 40 mm for the holotype), and actually larger than most complete fossil eggs found so far, only smaller than the egg described from the upper Miocene of Pakistan [55]. Egg size is in line with egg sizes of extant forms and for values on the egg sizes and shell thickness of extant crocodylomorphs, (see [9], Table 3 therein). The EI (1.75) is the same as for the egg reported from Early Cretaceous Glen Rose Formation [47] and eggs from the Late Cretaceous of South America have a higher EI, but are much smaller [7,53]. In modern crocodylomorphs, only the eggs of *Crocodylus mindorensis* and *Crocodylus novaeguinae* have a higher average EI, respectively 1.86 and 1.77 [9]. The smooth outer surface is characteristic as in most other fossilized crocodylomorph eggshells, except for an egg from the upper Miocene of Pakistan that shows ornamentation [55]. The interstices between shell units are smaller than in *K*. *helleri* and *K*. *wilsoni*, the other oospecies in the oogenus *Krokolithes* [23,87], and the shell units wider than taller, contrarily to what is observed in modern crocodylomorphs which have wide inter-basal knob spaces and narrower and taller shell units. On the eggshell thickness, *Krokolithes dinophilus* (≈ 170–250 μm) is thinner than *Krokolithes wilson*i and *Krokolithes helleri* [87,88] with values closer to eggshells from the Cretaceous of France, Spain, Bolivia and Brazil [7,40,41,53]. The horizontal accretion lines are more pronounced in *K*. *dinophilus* than in other *Krokolithes* specimens, closer to what is observed in *A*. *mississipiensis* eggshells [9].

## Discussion

### Egg taphonomy and eggshell preservation

Considering the exceptional preservation of FCT-UNL706, uncrushed, and the extreme fragility of the eggshell, the unhatched clutch (Fig 4) was most likely buried *in situ*, where the oviposition occurred. Contrarily, the eggshells from Paimogo (ML760, ML1795), Peralta (ML195), and Casal da Rola (ML1194), are fragmented, in some cases showing a marked sub-horizontal fracturing (Fig 6B), and, in the case of the eggs ML760 (Fig 4) and ML1795 (Fig 8A), showing clear signs of post burial damage, namely vertical compression, as the eggs are flattened and crushed.

The tabular arrangement of crocodile eggshell is evident at both microstructural (growth lines) and ultrastructural (tabular ultrastructural) levels in most fossil and extant taxa, although such features can be obliterated during fossilization [87]. By comparing the radial sections of *K*. *dinophilus* (Fig 6), with the extensively studied *A*. *mississipiensis* (see Fig 11 in [9]), the tabular arrangement within the shells are remarkably similar. On the other hand, in *S*. *portucalensis* (Fig 5C) no such organization is visible, but it is impossible to assess if this is a preservation artifact or an original feature of the eggshells. Despite this, *S*. *portucalensis* shows enough distinct crocodyloid characters, like ellipsoid eggs (EI of 1.62), slightly ornamented external surface, trapezoidal interlocking shell units widening from the basal knobs to the exterior to confidently place them within Krokolithidae.

### Clutch and egg size as proxies of female adult size

The holotype of *S*. *portucalensis* has the highest number of eggs on record for any single clutch in the fossil record, with 13, as well as the second smallest crocodylomorph eggs, only slightly bigger than those associated to the remains of *Yacarerani boliviensis*, from the Late Cretaceous of Bolivia [53]. On the other hand, and considering only complete eggs, the holotype of K. *dinophilus* is one of the largest crocodiloid fossil eggs known, 70 x 40 mm, in the same size range of the eggs of *A*. *mississipiensis*.

**Fig 10:**
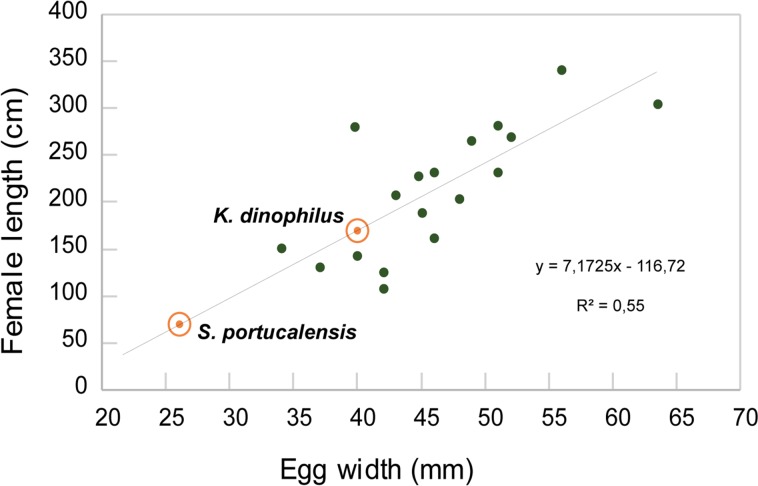
Relationships between egg width and female length in modern crocodylians and Portuguese ootaxa. Adult female mean length values from [2]. Egg width values from ([9] and references therein). Regression line calculated for 19 extant species of crocodylomorphs. Regression equation used to calculate average female length of egg layer taxa for *Suchoolithus portucalensis* and *Krokolithes dinophilus*, shown in the graphic.

Reproductive allometry studies on extant crocodylomorphs and, more recently, on *Diplocynodon darwini* from the Middle Eocene of Geiseltal, or known skeletal remains-egg associations, indicate a correlation between body size and egg mass [2,10,53,89,90]. Estimating egg mass is difficult in fossil eggs, but a correlation between body length and egg width–the constraining dimension on egg size that can be related to the size of the oviduct and thus, with the size of the female [89,91]–can be established (Fig 10). Considering that the allomeric relation, as in previous studies [2,10,89,90], is loosely supported (R^2^ = 0.55), an approximate size of 70 cm for the egg layer of *Suchoolithus* and 170 cm for the egg layer if *K*. *dinophilus* can be estimated. Thus, the important differences seen in egg size between the two ootaxa of the Lourinhã Formation provides further evidence on the coexistence of different sized crocodylomorphs, probably occupying different ecological niches in the Late Jurassic ecosystems of Portugal, a hypothesis also supported by the skeletal record [92–95]. In fact, similar sympatry can be observed in the Late Jurassic of France and Germany, extending geographically what has been reported by Tennant and Mannion [96].

### Comparison with the skeletal record

None of the eggs in this study have provided up to this point embryonic material or associated hatchlings that might provide definitive evidence that could allow ascribing these finds to a specific crocodylomorph taxon. Also, the morphological stability of the eggshells makes the association of the egg material to a specific crocodylomorph taxon extremely difficult, more so as the paleodiversity increases. However, the remains of crocodylomorphs in the Lourinhã Formation are abundant and the diversity of forms high, with well documented ocurrences, which might at least help in narrowing the putative egg layer. The following taxa are known from the Lourinhã Formation: *Bernissartia* sp., cf. *Alligatorium*, *Goniopholis baryglyphaeus* Schwarz 2002, *Machimosaurus hugii* von Meyer 1837, *Lisboasaurus estesi* Seiffert, 1973, *Lusitanisuchus mitrocostatus* Schwarz & Fechner 2004, *Theriosuchus guimarotae* Schwarz & Salisbury 2005 [92–106]. Egg size can also provide help to narrow the identification of the putative egg layers of both *S*. *portucalensis* and *K*. *dinophilus*. The eggs of FCT-UNL706 are very small which would seem to suggest that the more likely candidate as parent taxa would be one of the small forms of crocodylomorphs (≤ 1 meter) from the Lourinhã Formation, such as *Bernissartia*, *Alligatorium*, *Lisboasaurus*, *Lusitanisuchus* or *Theriosuchus* [92–95]. On the other hand, *K*. *dinophilus* would be relatable to a medium sized crocodylomorph (2–3 meters), such as *Goniopholis*, a neosuchian ubiquitous throughout the Late Jurassic [92,104].

### Crocodylomorph oodiversity through time

Adding these two new ootaxa, the number of valid crocodylomorph ootaxa is five oospecies distributed among three valid oogenera, with only one of those not included in Krokolithidae, *M*. *kohringi* [31]. This contrasts with the much more diverse eggshell morphotypes attributed to dinosaurs. The reason for such a morphological conservatism among crocodiloid eggs is not yet well understood, but Moreno-Azanza et al. [107] briefly addressed this issue. These authors postulated that the contrast between the high diversity observed within the lineage of Crocodylomorpha in the past and the reduced modern representation of the clade, the low diversity of the eggs, and the absence or scarce record of gravid specimens or fossil embryos may raise the issue of a possible bias in the classification and proposed evolution of this morphotype. Nevertheless, our data shows that the eggshell structure related to modern crocodyloids was present and may have been ubiquitous among crocodylomorph taxa as early as the Late Jurassic.

### Implications of the occurrence of *K*. *dinophilus* with theropod eggs

The discovery of *K*. *dinophilus* associated with theropod nests and eggshells raises some still unresolved questions. The occurrence of *K*. *dinophilus* with theropod eggshells belonging to the same, or at least very closely related ootaxa could be indicative of some type of relationship between the two. ML760 and ML1795 were found associated with the Paimogo nest, attributed to *Lourinhanosaurus* [58–61,64]. ML1194 was found with theropod eggshells that are closely related, if not the same ootaxon, to the Paimogo eggshells and to *Preprismatoolithus coloradensis* [64]. The latter is attributed to *Allosaurus* [108]. ML195 was also recovered from an unidentified theropod nest.

The absence of a modern analog of these occurrences only allows for speculative considerations regarding a putative relationship between theropods and crocodylomorphs in the Late Jurassic of Portugal. Extant crocodylomorphs are the top predators in many ecosystems, and even smaller genera, more likely to be vulnerable to predators, are usually more reclusive and more heavily armored than their larger counterparts, therefore effectively decreasing the risk of predation [108]. In the Late Jurassic, this was not the case since the top tiers of the terrestrial food chain were occupied by a range of medium- to large-size theropods [72,73]. Nowadays, crocodilian nesting sites are located preferentially in secluded, marginal areas, frequently watched over by a parent, and so nesting sites of potential predators nearby are highly unlikely. These aspects of crocodilian behavior are, according to Somaweera and colleagues [109], likely to have evolved as a response to predatorial risk. Furthermore, the highest rates of predation on modern crocodilians occur on the earliest stages of life (i.e. eggs and hatchlings), with small mammals and varanids as potential predators [109]. It would be plausible to assume that the risk was even higher in the Late Jurassic due to the higher number of potential predators, even for a medium-sized taxon like the one that probably laid *K*. *dinophilus* eggs, as previously discussed.

A significant drawback is the lack of a more complete record, in the sense that, besides the Paimogo eggs, the material is fragmented and suggests that it might have been transported and posteriorly deposited, therefore not in its original nesting context. It is premature to make any conclusions without more unequivocal evidence. Thus, what the association of the same crocodylomorph ootaxon with apparently the same theropod ootaxa (and probably same theropod taxa) means is still a mystery, but it is a fact that should not be ignored. Going forward, further findings and studies are needed to ascertain if there was indeed some kind of reproductive relationship between crocodylomorphs and theropods in the Late Jurassic of Portugal and possibly develop a new perspective on unknown reproductive strategies and behavior of the Crocodylomorpha.

## Conclusions

The present study confirms the presence of eggs and eggshells of crocodylomorphs in the Late Jurassic Lourinhã Formation of Portugal. These findings represent the oldest recovered to date, extending back the range of crocodylomorphs eggshells by 7 Ma.

A new oogenus and two new oospecies are erected. *Suchoolithus portucalensis* oogen. et oosp. nov, differs from other Krokolithidae eggs by having small egg size, thin eggshell, and very tightly packed shell units with no interstices between neighbouring shell units. *K*. *dinophilus* differs from other Krokolithes oospecies by the larger eggs, smaller shell thickness, and low porosity.

The diversity within Krokolithidae is then increased and the number of crocodiloid ootaxa is now five, namely 3 oogenera and 5 oospecies. Additionally, we verified and confirmed that the basic crocodiloid eggshell structure has shown a morphological conservatism over a period of 150 Ma.

The lack of associated skeletal remains precludes a taxonomic identification of the eggs, although the differences in size allow to narrow down plausible egg layers by correlating with the known Crocodylomorpha of the Lourinhã Formation. *S*. *portucalensis* was probably laid by one of the several small crocodylomorph taxa, but *K*. *dinophilus* was probably laid by a medium size form, such as *Goniopholis*.

## Supporting information

S1 Table Occurrences of fossil crocodylomorphs eggs and eggshells. (PDF)
