# Supplementary material for: Two new ootaxa from the late Jurassic: The oldest record of crocodylomorph eggs, from the Lourinhã Formation, Portugal
Source: PLoS One. 2017 Mar 8;12(3):e0171919. doi: 10.1371/journal.pone.0171919 (PMC5342183; doi:10.1371/journal.pone.0171919)
Supplement: S1 Table — (PDF) [file pone.0171919.s001.pdf]

Two new ootaxa from the Late Jurassic: the oldest record of crocodylomorphs eggs, from the Lourinhã Formation, Portugal

Supporting Information 1

S1 Table. Fossil crocodylomorph eggs and eggshells.

Here are presented the fossil occurrences of eggs and eggshells of crocodylomorph affinity, as per the parataxonomical standard used for this type of eggshell (i.e., Krokolithidae). **L:** egg length; **W:** egg width; **ST:** eggshell thickness. The values in each column are given by the authors in the corresponding reference. Other discrepancies in the amount of information presented in the table are simply caused by lack of that information in the original reference.

Reference	L (mm)	W (mm)	ST (μm)	EI	Age & location
Patnaik & Schleich, 1993	64	54	190-660	1.19	Pliocene, Upper Siwaliks, Moginand, Himachal Pradesh, India
Panadés I Blas & Patnaik, 2009	84	64-54	180-760	1.31-1.56	Late Miocene, Chinji Fm, Dhok Yakoob, Uchhri, Pakistan
Kohring, 1992	-	-	300	-	Early Miocene, Lower Freshwater Molasse, Ulm, Baden-Württenberg Germany
Vianey-Liaud *et al.*, 2014	-	-	330-450	-	Late Oligocene, Saint-Privat-des-Vieux, Gard, France
Kohring & Hirsch, 1996	53	30	-	1.77	Middle Eocene, lignite beds (“Obere Mittelkohle”), Geiseltal, Halle, Germany
	52	25	300-450	2.08	
	60	35	350-450	1.71	
	40	19	290-360	2.11	
	35	30	300	1.17	
	44	44	360-450	1.00	
Hastings & Hellmund, 2015	68-66	43-37	-	1.79-1.53	Middle Eocene, coal beds (Oberes Hauptmittel), Geiseltal, Halle, Germany
Hirsch & Kohring, 1992	68-65	44	600-700	1.48-1.55	Middle Eocene, Bridger Fm, Wyoming, USA
Hirsch, 1985	56 (50)	36 (30)	250-450	1.67	Eocene, DeBeque Fm, Parachute, Colorado, USA
Singh *et al.*, 1998	-	-	350	-	Late Cretaceous (Maastrichtian), Intertrappean Beds, Malabar Hill, Bombay, India
Prasad *et al.*, 2015	-	-	420-480	-	Late Cretaceous (Maastrichtian), Intertrappean Beds, Kislapuri, Madhya Pradesh, India
Srivastava *et al.*, 2015	68	44	430-470	1.54	Late Cretaceous (Maastrichtian), Lameta Fm, Lameta Ghat, Madhya Pradesh, India
Jackson & Varrichio, 2016	-	-	422-410	-	Late Cretaceous (Maastrichtian), Hell Creek Fm, Garfield County, Montana, USA
Moreno-Azanza *et al.*, 2013	-	-	750	-	Late Cretaceous (Maastrichtian), La Posa Fm, Arén, Huesca, Spain
Kerourio, 1987	-	-	640	-	Late Cretaceous (Maastrichtian), Bouches-du-Rhônes, Aix-en-Provence, France
Garcia, 2000	-	-	290	-	Late Cretaceous (Maastrichtian), La Neuve, Aix-en-Provence, France
Marsola *et al.*, 2016	73-47	48-29	130-150	1.71-1.35	Late Cretaceous (Maastrichtian), Campina Verde, Minas Gerais, Brazil
Ribeiro *et al.*, 2006	45-50	35-30	240-360	1.28-1.67	Late Cretaceous, Araçatuba Fm, Marília, São Paulo, Brazil
Oliveira *et al.*, 2011	58-65	32-36	150-250	1.81	Late Cretaceous, Adamantina Fm, Jales, São Paulo, Brazil
Novas *et al.*, 2009	30	16	200	1.88	Late Cretaceous (Turonian-Santonian), Cajones Fm, Santa Cruz de La Sierra, Bolivia
Rogers, 2000	49	28	600-700	1.75	Early Cretaceous (early Albian), Glen Rose Fm, Erath County, Texas, USA
Buscalioni *et al.*, 2008	-	-	250	-	Early Cretaceous (upper Barremian), La Huérgina Limestone Fm, Cuenca, Spain
Kohring, 1990	-	-	300-700	-	Early Cretaceous (early Barremian), Galve, Teruel, Spain
Moreno-Azanza *et al.*, 2015	-	-	524	-	Early Cretaceous (early Barremian), Blesa, El Castelar and Mirambel Fm, La Cantalera, Teruel, Spain
Canudo *et al.*, 2010	-	-	300	-	Early Cretaceous (late Hauterivian-early Barremian), Blesa Fm, La Cantalera, Teruel, Spain
Ensom, 1997, 2002	-	-	150-400	-	Early Cretaceous (Berriasian), Cherty Freshwater Mb., Lulworth Fm. (Purbeck facies), Dorset, UK
Grellet-Tinner, 2010	-	-	200	-	Early Cretaceous (Berriasian), Cherves-de-Cognac, Charente, France
Antunes *et al.*, 1998	70	40	200-350	1.75	Late Jurassic (late Kimmeridgian), Praia Amoreira-Porto Novo mb., Lourinhã fm, Paimogo., Lourinhã, Portugal
	42	26	163	1.62	Late Jurassic (Tithonian), Assenta mb., Lourinhã fm., Cambelas, Torres Vedras, Portugal
This study	-	-	239 (253)	-	Late Jurassic (late Kimmeridgian-early Tithonian), Praia Azul mb, Lourinhã fm, Peralta, Lourinhã, Portugal
	-	-	200 (220)	-	Late Jurassic (late Kimmeridgian-early Tithonian), Praia Azul Mb, Lourinhã fm, Casal da Rola, Lourinhã, Portugal
	-	-	172 (243)	-	Late Jurassic (late Kimmeridgian-early Tithonian), Praia Azul mb,, Lourinhã fm., Paimogo, Portugal
	70	40	248 (392)	1.75	Late Jurassic (late Kimmeridgian), Praia Amoreira-Porto Novo mb., Lourinhã fm., Paimogo, Lourinhã, Portugal
